# Wire cerclages as part of osteosynthesis- examination for optimal placement

**DOI:** 10.1007/s00402-025-05795-y

**Published:** 2025-04-05

**Authors:** Denis Visser, Christopher Bliemel, Thomas Schürholz, Rene Aigner, Steffen Ruchholtz, Martin Bäumlein

**Affiliations:** 1 Universitätsklinikum Gießen und Marburg, Marburg, Germany; 2 Philipps University of Marburg, Marburg, Germany

**Keywords:** Cerclage, Osteosynthesis, Load to failure, Operative skill, Clinical application, Biomechanical aspects, Technical aspects

## Abstract

Wire cerclages are commonly used during osteosynthesis of bone shaft fractures. To date, there is no study that examines the intraoperative utilization by different experienced surgeons in terms of reproducibility. This study aimed to test the hypothesis that a double-looped-cerclage is superior to a single-looped-cerclage in terms of reproducibility and uniform contact pressure. 27 medical doctors working in orthopedics/trauma surgery took part in this study. A wire cerclage was applied to a bovine bone half-shell model mounted on a dynamometer. A single-looped-cerclage and a double-looped-cerclage were applied alternately 5 times each. The applied force before modelling the cerclage knot on the bone (fbM) and the applied force after modelling on the bone (faM) were recorded in a blinded manner. The median faM in a double-looped-cerclage was 375 N (IQR 230–531 N) and therefore significantly higher (*p* < 0.05) than in a single-looped-cerclage (150 N (IQR 83–232 N)). As a result of applying the cerclage knot to the bone, the force decreased by an average of 80 N, with no differences in single-looped-cerclage or double-looped-cerclage. (Force-loss single-looped-cerclage 84 N (IQR 35.75–132 N); Force-loss double-looped-cerclage 82 N (IQR 46–116 N). The quartile dispersion coefficient as an expression of dispersion for the 5 applications each was 0.7121 (IQR 0.6544–0.8979) for single-looped-cerclage and was significantly higher than for double-looped-cerclage 0.3876 (IQR 0.2376–0.5184). In summary, this study showed that a double-looped-cerclage was superior to a single-looped-cerclage when used intraoperatively in terms of contact pressure and reproducibility.

## Highlights

The double looped cerclage is significantly superior to a single looped cerclage after the cerclage knot has been tied to the bone.

When knotting and modelling the single looped cerclage as well as the double cerclage to the bone, the pressure force is reduced.

The quartile dispersion coefficient as an expression of dispersion showed no significant difference between residents and consultants.

## Introduction

The application of a wire cerclage as part of an osteosynthesis of a bone shaft fracture is a frequently used method [1–3]. It is known that a cerclage can help to fix fracture fragments in the restored physiological position and is therefore of great importance as a repositioning tool [4]. Various influencing factors have already been examined in biomechanical studies, some of which have shown a significant difference in relation to the stability of osteosynthesis. The diameter of the cerclage, the application technique, the technique of cutting the cerclage and the direction of bending to attach it to the bone were examined as influencing factors [5–8]. It was also described that a force of at least 200 N should be used when applying the cerclage, as this showed the best results in terms of the longevity of a cerclage [9]. 

One of the disadvantages discussed in the literature is the potential impairment of periosteal blood flow caused by the tension and pressure of the applied cerclage. The periosteum plays a crucial role in bone healing as it supplies blood flow and osteogenic progenitor cells. Impaired periosteal blood flow could theoretically delay or hinder fracture healing. While some animal studies report a significant reduction in periosteal blood flow due to cerclage application, other studies, including investigations on human cadaveric femora, found no clinically relevant impairment [10–12]. Although definitive evidence is lacking, concerns remain about the potential risks of cerclage application to the bone’s vascular supply, warranting further investigation.

To our knowledge, no previous study has specifically investigated the application technique of wire cerclages by surgeons with varying levels of experience in terms of reproducibility and effectiveness. This study aimed to test the hypothesis that double-looped cerclages are superior to single-looped cerclages in terms of applied force, reproducibility, and uniformity of contact pressure during application.

## Methods

### Experimental setup

**Fig. 1:**
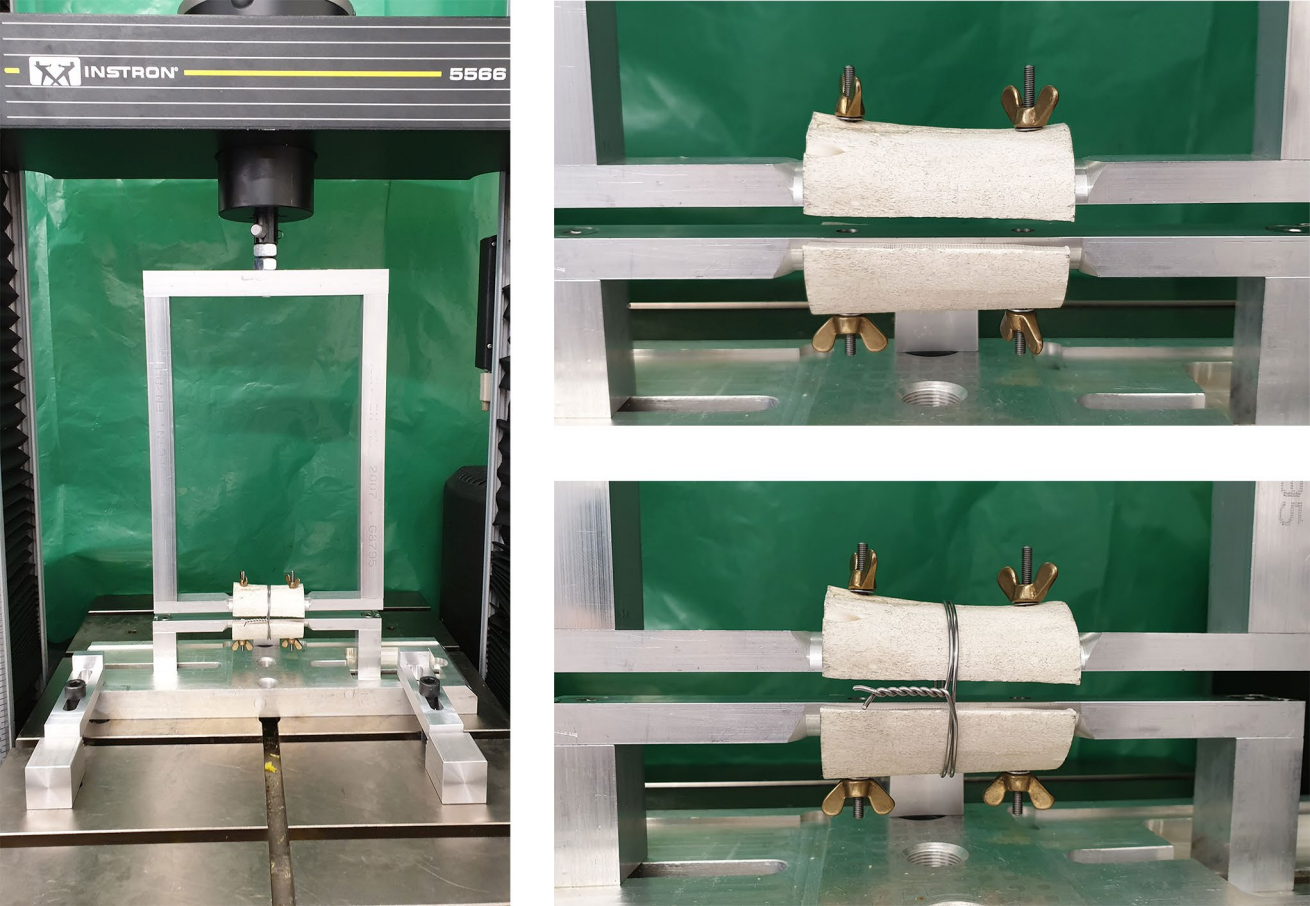
Experimental setup. The two halves of the tubular bone are mounted on the measuring frame with a small gap in between to measure the force applied by the cerclage

A bovine tubular bone was drilled intramedullary and cut into two halves. These halves were each mounted on a half cylinder of the measuring frame, which was clamped via the dynamometer attachment in the Instron 5566 biomechanical loading apparatus (Instron Cor., Darmstadt, Germany). There was a gap in between both halves of 1 mm to measure the force applied by the cerclage. The bone used should ensure a setting that is as realistic as possible regarding friction forces of the cerclage (Fig. 1).

### Participants

27 medical doctors working in orthopedic/trauma surgery took part on a voluntary basis. All participants received an explanation about the study and provided written informed consent. The training status - consultant or resident doctor and gender were recorded. Based on a power of 0.80 and an alpha error of 0.05, a sample size of 13 was calculated. The study was approved by the ethics committee.

### Experimental protocol

A 1.25 mm wire cerclage (DePuy Synthes GmbH, Solothurn, Switzerland) was placed a total of 10 times per participant, 5 times as a single-looped-cerclage and 5 times as a double-looped-cerclage. Single-looped and double-looped-cerclage were applied alternately, with the start being decided by a coin toss. The applied force of the cerclage to the bone before and after modelling the cerclage knot on the bone was measured in a blinded manner. The quartile dispersion coefficient was calculated for each participant to measure the consistency for applying a single-looped-cerclage and double-looped-cerclage.

### Statistical analysis

Statistical analysis was carried out using GraphPad Prism (Version 6.01, GraphPad Software Inc., San Diego CA, USA). The individual evaluations were first checked for standard normal distribution using the D’Agostino-Pearson test. Significant differences between the individual groups were tested using Kruskal Wallis tests and Dunn’s multiple comparison tests for independent samples. Statistical significance was set at *p* < 0.05.

## Results

27 medical doctors working in orthopedic/trauma surgery took part at the study. 9 were female and 18 were males. In terms of training, 13 were resident doctors and 14 were consulting physicians.

The median applied force before modelling the cerclage knot on the bone differed significantly (*p* < 0.05). The median force of the single-looped-cerclage was 150 N (IQR 83–232 N) whereas the median force of the double-looped-cerclage was 375 N (IQR 230–531 N).

**Fig. 2:**
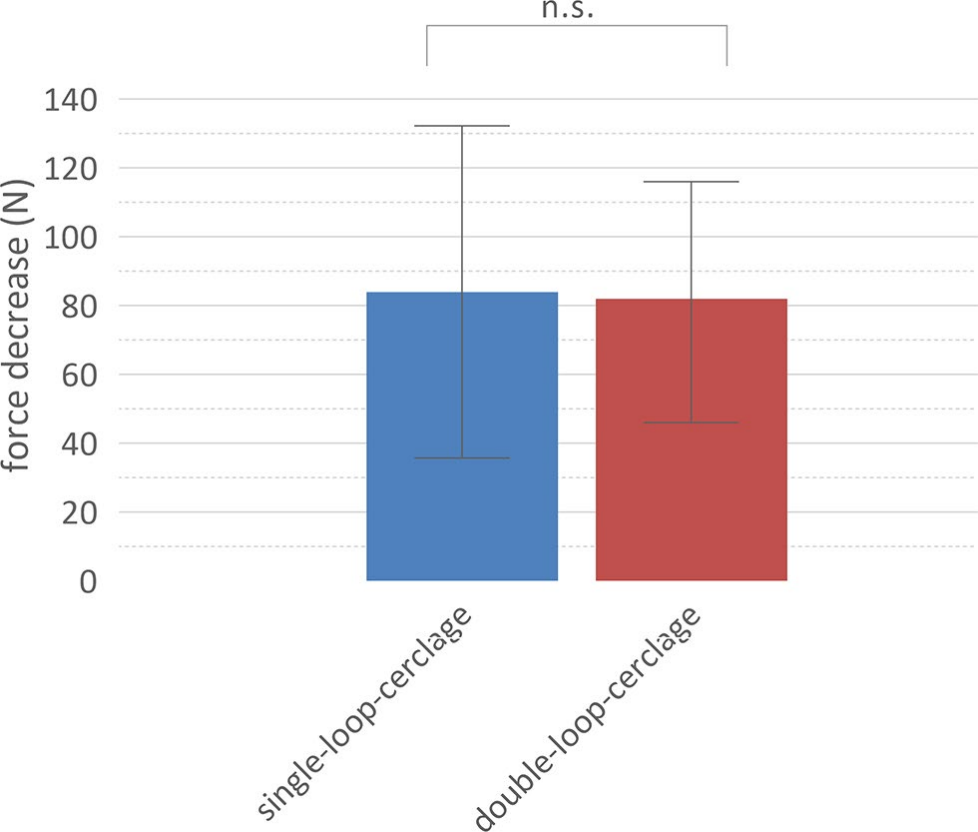
Decrease in the applied force during application. There is no significant difference in the decrease of the applied force during applying the cerclage knot to the bone. Median and interquartile range are displayed

A decrease in the applied force could be observed during applying the cerclage knot to the bone. There was no significant difference between single-looped and double-looped-cerclage. The force decreased by 84 N (IQR 33.75–132.3 N) for the single-looped-cerclages and by 82 N (IQR 46–116 N) for the double-looped-cerclages (Fig. 2).

**Fig. 3:**
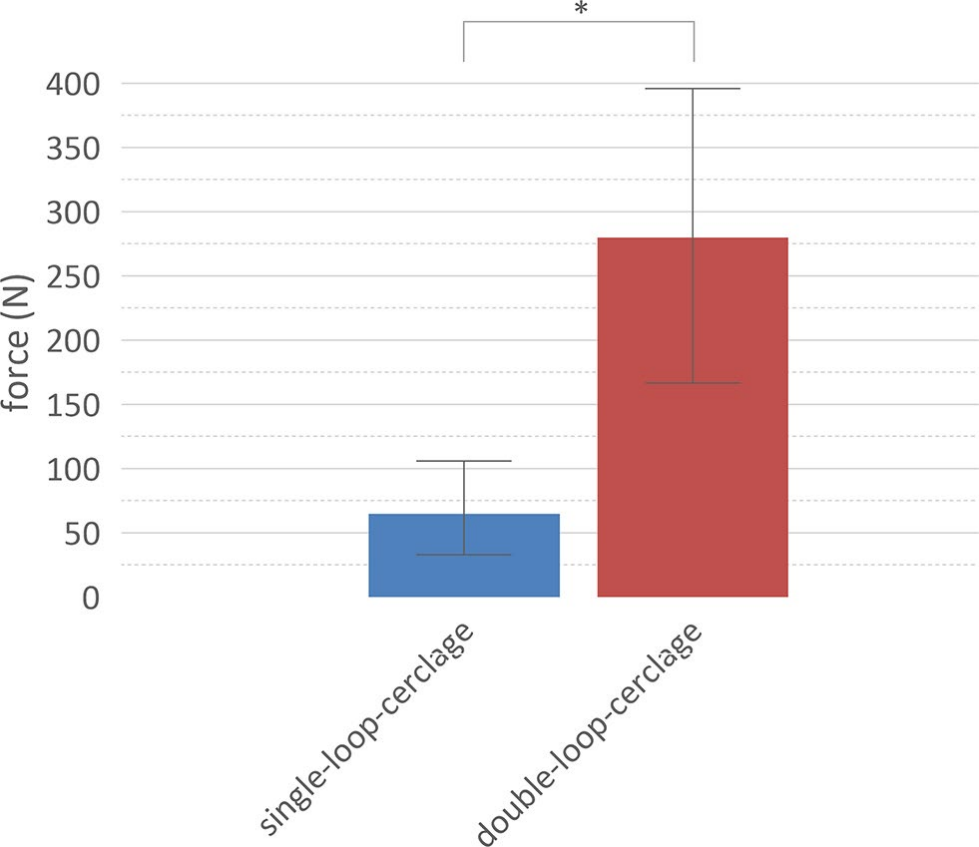
Applied force after modelling the cerclage knot to the bone. There is a significant difference in the applied force after modelling the cerclage knot to the bone. Median and interquartile range are displayed

The difference between single-looped and double-looped-cerclage and the applied force after modelling the cerclage knot to the bone was significant (*p* < 0.05). The median force for the single-looped-cerclages was 65 N (IQR 33–106 N) and 280 N (IQR 167–396 N) for the double-looped-cerclages (Fig. 3).

**Fig. 4:**
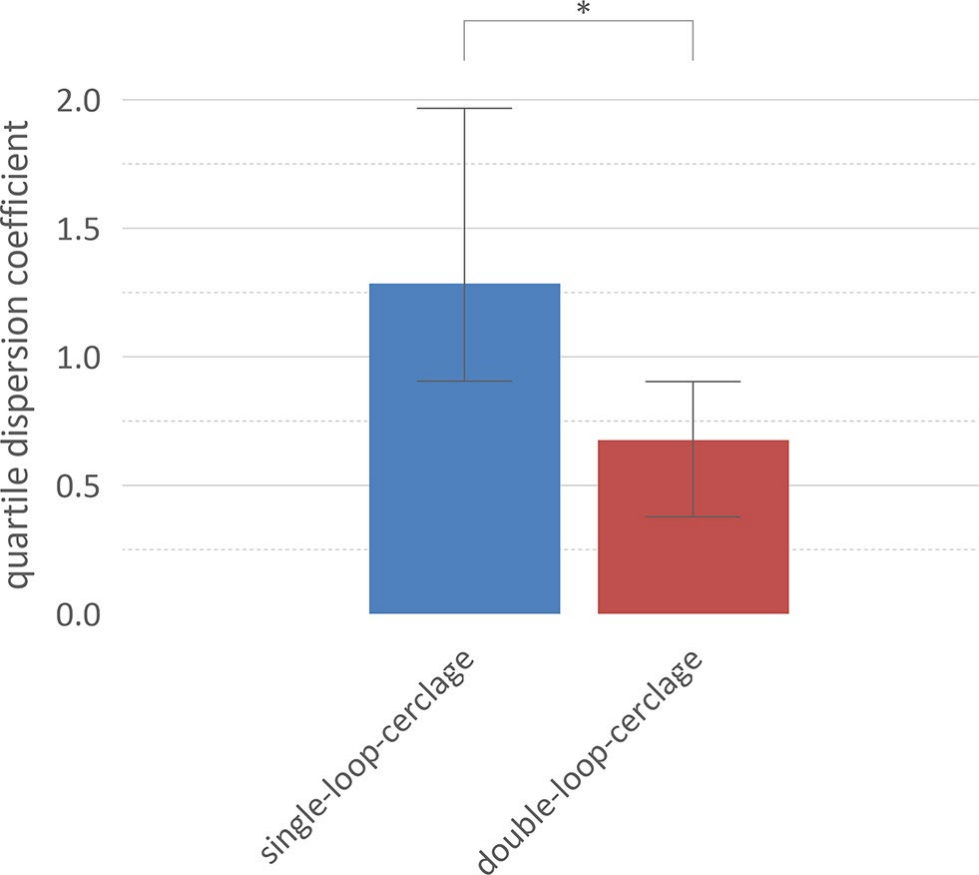
Intraoperative dispersion during application. The intraoperative dispersion during application is significant better for double-loop-cerclages compared to single-loop-cerclages. Median and interquartile range are displayed

There was a difference of the force after applying the cerclage knot of resident doctors and consulting physicians, but it was not significant. (Single-looped-cerclage resident doctors: 43 N (IQR 27–84 N); Single-looped-cerclage consulting physicians 84 N (IQR 51–127 N). Double-looped-cerclage resident doctors: 222 N (IQR 127–335 N); Double-looped-cerclage consulting physicians 335 N (IQR 233–461 N)) (Fig. 4).

For each proband, the quartile dispersion coefficient was calculated for the 5 runs of each experiment as an expression of the dispersion. A statistically significant difference was observed (*p* < 0.05). (single-looped-cerclage- median 1.286 (IQR 0.9063–1.967); double-looped-cerclage- median 0.6775 (IQR 0.3786–0.9039). There was no significant difference regarding resident doctors and consulting physicians.

## Discussion

Many studies examined the load-to-failure of cerclages. It was shown that the double-looped cerclage withstood a higher tensile strength than a single-looped cerclage [1, 4–6, 13]. However, there are only few studies that examined the initial force applied when comparing single-looped-cerclage and double-looped-cerclage. *Lenz et al.* investigated the biomechanical deformation of different cerclages and cable cerclages. Similar to our study, they were able to determine a higher pretension of the double-looped-cerclage compared to the single-looped-cerclage [2].

Furthermore, this study showed that there is a decrease in strength when the cerclage knot is modelled onto the bone. Double-looped-cerclages and single-looped-cerclages showed a similar decrease of force of around 80 N. *Waehnert et al.* examined, among other things, the pretension force when applying different cerclages. For a 1.25 mm single-looped cerclage, identical to the one we used in this study, which was applied 5 times, they were able to see a decrease in force from an initial 158 N (SD 8 N) to 74 N (SD 9 N) after modelling the cerclage knot on the bone [7]. These results are consistent with our findings.

A key concern regarding cerclage application is the potential impairment of periosteal blood flow due to tension and pressure. Some studies suggest that cerclage application may reduce periosteal blood flow, theoretically delaying or hindering fracture healing [10, 11]. However, other studies, including investigations on human cadaveric femora, found no clinically relevant impairment of blood supply caused by cerclage application [10, 12].

Notably, no studies have specifically investigated differences in periosteal blood flow impairment between single- and double-looped cerclages. While double-looped cerclages generate higher compression forces and exhibit greater application consistency, as indicated by the lower dispersion in our study, it remains unclear whether the increased force might negatively impact periosteal blood flow. *Apivatthakakul et al.* demonstrated that percutaneous cerclage wiring does not significantly disrupt femoral blood supply, suggesting that certain cerclage techniques may be safer than previously assumed [10]. Similarly, *Wang et al.* reported no adverse effects on bone healing in patients treated with cerclages [12].

To our knowledge, there is no study that puts the surgeon’s experience in the context of cerclage placement. This study was able to show that although there is a small difference with a tendency for consulting physicians to apply the cerclage with a higher force than resident doctors, these were not significant.

The application of a cerclage in osteosynthesis is described as a relatively simple method [4, 11, 14].

In this study it was found that the force applied to the bone due to the cerclage is inconsistent even when the same surgeon performs the procedure. However, it was shown that the double-looped-cerclage is applied more consistent and with lower dispersion than the single-loop-cerclage and should therefore be preferred.

### Limitations

Since the present analysis is based on an ex vivo setting, some limitations must be taken into account. There was a bone-model used to apply a cerclage. In a regular surgical site, it can be assumed that poor visibility, difficult soft tissue or anatomical conditions may complicate the correct application of a cerclage.

Since both bone half-shells were clamped in a fixed frame, the anatomical conditions associated with an unstable fracture that has to be fixed using a cerclage were represented inadequately.

## Conclusion

This study demonstrated that double-looped cerclages are superior to single-looped cerclages in terms of both applied compression force and reproducibility during placement. Specifically, double-looped cerclages achieved significantly greater compression forces after knot application and exhibited lower dispersion in force consistency across repeated applications.

Furthermore, while both cerclage types experienced a similar reduction in applied force when modeling the knot onto the bone, double-looped cerclages maintained significantly higher forces after this process. These findings suggest that double-looped cerclages offer better performance and reliability in an ex vivo surgical setting and may be considered the preferred technique for achieving consistent and effective cerclage application.

## Data Availability

No datasets were generated or analysed during the current study.
